# Foaming and Other Functional Properties of Freeze-Dried Mare’s Milk

**DOI:** 10.3390/foods12112274

**Published:** 2023-06-05

**Authors:** Dorota Cais-Sokolińska, Joanna Teichert, Jolanta Gawałek

**Affiliations:** Department of Dairy and Process Engineering, Faculty of Food Science and Nutrition, Poznań University of Life Sciences, ul. Wojska Polskiego 31/33, 60-624 Poznan, Poland; joanna.teichert@up.poznan.pl (J.T.); jolanta.gawalek@up.poznan.pl (J.G.)

**Keywords:** mare’s milk, freeze-drying, functional food

## Abstract

The aim of this study was to evaluate the effect of the freeze-drying process on the preservation of mare’s milk. This was achieved through the characterization of the functional properties of reconstituted freeze-dried mare’s milk. The chemical composition, bulk density, foam capacity, and ability to form emulsions of the atherogenic, thrombogenic, and hypercholesterolemic fatty acid index were investigated. The freeze-drying process did not change the proportion of the milk components in the dry matter. The moisture content of the freeze-dried mare’s milk was 10.3 g/kg and the bulk was below 0.1 g/mL. The foaming capacity was 111.3%; hence, the foaming capacity of the milk was very poor. The oil binding capacity was 2.19 g/g of protein. The freeze-drying process improves the binding degree and retention of oil by milk proteins, but produced foam was unstable, short-lived, and lacked the ability to retain air fractions. The atherogenic index and thrombogenic index values calculated for reconstituted milk were 1.02 and 0.53, respectively. The hypercholesterolemia fatty acid index was 25.01.

## 1. Introduction

Mare’s milk has gained popularity in recent years due to its nutritional value and ease of digestibility, which are related to its structural and functional properties, and highlight its potential use in human nutrition [1]. Its biochemical and biophysical properties are similar to that of human milk, especially in regard to high albumin content compared to casein. Additionally, high whey protein and essential amino acid levels benefit humans. Hence, mare’s milk belongs to the albumin group, whereas domestic animal milk (except for donkeys) belongs to the casein group [2,3]. Importantly, proteins and peptides of mare’s milk, such as lysozyme and lactoferrin, play an influential role in the overall antimicrobial activity [1,4]. Lysozyme synergistically works with lactoferrin and immunoglobulins in antimicrobial activity [2,5]. Lactoferrin displays several biological activities, such as defense against infection by microorganisms and anti-inflammatory activity [6]. It has been reported that lactoferrin can act as a natural barrier to counteract COVID-19 infection, reversing iron disorders, and modulating the immune response by down-regulating pro-inflammatory cytokines [7]. The health quality of milk is additionally determined by the fatty acids profile. Mare’s milk fat is characterized by a high level of polyunsaturated fatty acids (PUFA) and a low level of saturated fatty acids (FA). The value of atherogenic (AI) and thrombogenic indices (TI) calculated on fatty acid composition qualifies mare’s milk as a choice for people with inflammatory conditions and also for reducing the risk of different diseases [3]. The availability of fresh mare’s milk on the consumer market is very limited owing to the small number of dairy mare’s farms and, in addition, the low yield of mares’ milk. According to reports, daily mare’s milk production is estimated to be 15–35 g kg^−1^ bodyweight (within 500–2000 mL) [8,9]. Therefore, the method of preservation, storage, and possible further use is a crucial element.

Freeze-drying is a known optimal drying method, especially when considering the preservation of heat-sensitive biological materials [10,11,12,13]. This approach allows for the microbiological stability and long shelf life of products due to low moisture content (<2%), short reconstitution (rehydration) time due to the retention of the material’s structure (low drying shrinkage), low weight, high recovery of volatiles and nutrients, and high viability and bioactivity level [10,11,12,13,14,15,16,17,18]. However, freeze-drying is not widely used in the production of common powdered dairy products due to high production costs compared to other drying methods [10,13,16,18,19]. In the case of the production of special-nutrition or health-promoting products based on milk with unique properties, freeze-drying is the most appropriate process. Although there are numerous studies on the freeze-drying process of cow [12], sheep [20], goat [21], camel [19,22], donkey [23], and human [14] milk, reports on the freeze-drying process of mare’s milk are rare [12,19,20,21,22,23]. To date, mare’s milk is usually offered as a fresh raw material, frozen, or in the form of fermented milk (kumys) [24,25]. A comprehensive understanding of freeze-dried mare’s milk is important from a consumer perspective for those who are looking for innovative products. At the same time, these products must be easy to prepare and consume, e.g., in the form of drinks or cocktails. Furthermore, health-friendliness is vital as it directly correlates with the health and well-being of the consumer [26]. Harizi et al. [27], in their own research and based on other scientific reports, indicated that the freeze-drying process, compared to spray drying, allows for greater preservation of the nutritional, bioactive, and antioxidant values of milk. Previous studies of cow [27,28], camel [27,29], goat [21], and human [30] milk have proven that low-temperature preservative treatment is more beneficial to the health-promoting values than treatment at higher temperatures used during spray drying. The freeze-dried process maintains the physical and chemical stability of the milk powder more during storage than spray drying. Preserving the health benefits of mare’s milk, such as the protein profile and lipid profile, is important in developing products for older consumers and those with lower immunity. Current data show that the increasing incidence of the COVID-19 virus in the senior age group highlights the need to develop new functional foods adapted to consumers with reduced immunity.

Considering the aforementioned issues, the presented study aimed to examine the physical properties (mainly foaming and emulsifying capacity) and pro-health properties (lipid profile index values) of reconstituted freeze-dried mare’s milk, which determine its potential use as an ingredient of food products, e.g., mare’s milk-based drinks.

## 2. Materials and Methods

### 2.1. Raw Mare’s Milk

Mare’s milk was collected from 6 Polish Coldblood mares reared on a mares dairy farm in the Wielkopolska region (Western Poland). Mares were between 5 and 8 years of age, with live weights between 590 and 810 kg. The mares were mechanically milked from both teats of the udder once a day in the evening after 3 h of physical separation from their foals. The mares were milked in the 4th month of lactation. It is a period of full stabilization of the composition and properties of the milk [3]. Bulk mare’s milk was collected according to ref. [31]. Bulk mare’s milk (from six mares) was further the raw material in the freeze-drying process. The entire experiment was repeated 6 times (*n* = 6).

### 2.2. Freeze-Drying and Reconstituted Freeze-Dried Mare’s Milk

Fresh mare’s milk was frozen in silicone molds (to form frozen milk discs 25 mm in diameter and 10 mm thick) to −40 °C (in freezer RLHE0845, Labcold Ltd., Chineham, UK) before the freeze-drying process. Each time for the freeze-drying process, 0.6 L of milk and 120 silicone molds were used. The freeze-drying process was carried out on a semi-technical scale using the CHWC-20A freeze-dryer (Lyo-Tech Sp.z o.o., Międzyrzecz, Poland) to simulate an effective industrial process. Freeze-drying occurred in a single layer of frozen milk discs. Throughout the process, the vacuum was kept at approximately constant in the range of 60–70 Pa, and the temperature of the heating plates was varied, starting at 90 °C and ending at 40 °C. The complete freeze-drying process was conducted for 16 h. The powders were stored in foil laminate sealed pouches at 3 ± 0.5 °C for further analyses, but not for longer than 2 weeks. Freeze-dried mares milk samples were stored protected from light. However, the measurements were made during short-term exposure to light (450 lx, f 590 cd, 120 lm; TES-1335, TES Electrical Electronic Corp., Taipei, Taiwan).

The tested samples were: raw, freeze-dried, and reconstituted freeze-dried mare’s milk. A total of 26.5 g of freeze-dried mare’s milk was dissolved in 250 mL of distilled water at 18 °C (*w*/*v*, milk dispersion; reconstituted milk). Each sample’s measurement was performed in triplicate.

### 2.3. Moisture Content, Bulk Density and Water Activity

The moisture content of the freeze-dried mare’s milk was analyzed using a halogen moisture analyzer HX204/M (Mettler Toledo, Columbus, OH, USA) at 80 °C. The composition was determined according to the method described by the American Dairy Products Institute [32]. The raw and reconstituted milk composition was determined using DairySpecFT (Bentley Instruments Inc., Chaska, MN, USA).

The density of the studied raw milk and reconstituted freeze-dried mare’s milk was measured using an areometer (Gomar, Warszaw, Poland) at 20 °C within the range of 1.015 to 1.045 g/mL. For the measurement of untapped bulk density of the freeze-dried mare’s milk, a Scott Volumeter (HLL GmbH, Langenhagen, Germany) was used.

The water activity was measured with an AquaLab Series 4TE instrument (Decagon Devices Inc., Pullman, WA, USA).

### 2.4. Foaming Properties

Foam whipping of 100 mL of reconstituted milk was conducted over 2.5 min and 13,100 rpm using a food processor with a whipper, KASIA MRK-12 (MPM agd S.A., Milanówek, Poland). The following was determined: foam performance using Equation (1), foam stability using Equation (2), foam swelling using Equation (3), and proportion of air fraction in foam (Φ) based on foam swelling using Equation (4):

(1) $$FP=\frac{A}{B}\times 100$$

(2) $$FS=\frac{C}{A}\times 100$$

(3) $$FSW=\frac{wd-wf}{wf}\times 100$$

(4) $$\Phi =\frac{FSW}{FSW+100}$$

where A is the volume (mL) of the foamed solution immediately after the end of foaming τ = 0, B is the initial volume (mL) of the solution used for foaming, C is the total volume (mL) of the foam after τ = 30 min, wd (g) is the weight of 10 mL of solution used for foaming, and wf (g) is the weight of 10 mL of foam. All measurements were conducted at 25 °C [21].

### 2.5. Oil-Binding Capacity and Emulsifying Property

The oil-binding capacity was measured as described by Yang and Zhao [33]. The emulsifying activity index (EAI) was determined according to Alamprese et al. [34] with some modifications. Kujawski refined virgin rapeseed oil (Bunge Polska sp. z.o.o., Kruszwica, Poland) was used for emulsion preparation. Emulsion stability indices (ESI) were calculated according to the protocol described by Sun et al. [35]. An H500 homogenizer (Pol-Eko-Aparatura, Wodzisław Śląski, Poland), centrifuge (model 260, MPW MED Instruments, Warsaw, Poland), and RayLeigh UV-1601 spectrophotometer (Beijing Rayleigh Analytical Instrument, Beijing, China) were used for the measurements.

### 2.6. Powder and Foam Microstructure Illustration

Freeze-dried milk and foams were evaluated by optical microscopy using ProteOne (Delta Optical, Minsk Mazowiecki, Poland), which was additionally equipped with a DLT-Cam PRO microscope camera (Delta Optical, Minsk Mazowiecki, Poland). Freeze-dried mare’s milk was deposited onto a glass slide surface and covered with a cover slip for observation under an optical microscope. Samples of foam formed from freeze-dried mare’s milk (reconstituted milk) were prepared for observation according to Chang and Hartel [36]. Observations were made at 100× magnification using a ProteOne semiplanachromatic objective (Delta Optical, Mińsk Mazowiecki, Poland).

### 2.7. Fatty Acid Profiles

The fatty acid profile was determined according to the methodology described by Cais-Sokolińska et al. [37]. The atherogenic index (AI; Equation (5)), thrombogenic index (TI; Equation (6)), and hypercholesterolemia fatty acid index (HcFA; Equation (7)) of fat milk samples were calculated using the following formulas [38,39].

AI = (C12:0 + 4 × C14:0 + C16:0)/UFA(5)

TI = (C14:0 + C16:0 + C18:0)/[(0.5 × MUFA) + (3 × n-3) + (0.5 × n-6) + (n-3/n-6)](6)

HcFA = C14:0 + C16:0(7)

where UFA: unsaturated fatty acids, MUFA: monounsaturated fatty acids. Additionally, the hypocholesterolemic/hypercholesterolemic ratio (h/H) was calculated [40].

The content of fatty acids with the hypocholesterolemia effect (DFA; Equation (8)) and hypercholesterolemia fatty acids (OFA; Equation (9)) was calculated according to ref. [41].

DFA = UFA + C18:0(8)

OFA = SFA − C18:0(9)

### 2.8. Statistical Evaluation

Statistica data analysis software version 13.3.0 (TIBCO Software Inc., Palo Alto, CA, USA) was used for calculations. Hypotheses were verified based on a level of significance of α = 0.05. Differences between samples were calculated using a one-way analysis of variance followed by Tukey’s HSD post hoc test for multiple comparisons.

## 3. Results and Discussion

### 3.1. Composition and Physical Properties of Freeze-Dried Mare’s Milk

The freeze-drying process of mare’s milk resulted in a 9.3-fold thickening of the solid non-fat to 822.5 g/kg, and fat to 158.1 g/kg. The moisture content of the freeze-dried mare’s milk was 10.3 g/kg. The total protein content of freeze-dried mare’s milk was 221.3 g/kg, of which 126.1 g/kg was casein. The dominant ingredient was lactose (601.1 g/kg). The proportion between protein and lactose was therefore 0.4, while the protein participation in solid non-fat was 3.7.

The bulk density was determined volumetrically, indicating that the structural uniformity of freeze-dried mare’s milk was below 0.1 g/mL. The observed low bulk density value was indicative of a loose and low-packed matrix. Freeze-dried mare’s milk was very loose and light. In comparison, Sulieman et al. [42] showed that the untapped bulk density of freeze-dried camel milk was 0.38–0.52, freeze-dried cow’s milk was 0.38–0.45, and commercial milk was 0.37. In contrast, the loose bulk density of milk protein concentrate MPC60 had a value of 0.34 g/mL [43].

The water activity of the freeze-dried mare’s milk was very low, 0.0537. Freeze-dried bovine and ovine milk water activity measured by Brożek et al. [44] was 0.07 and 0.05, respectively. In contrast, the water activity of freeze-dried human milk obtained by Alves et al. [45] was much larger, at 0.3921. In comparison, the water activity freeze-dried yoghurt powders were between 0.211 and 0.258 [46]. Such significant differences in water activity may result from the porous structure of the sample. The greater the porosity, the greater the removal of the unbound water, which ultimately results in less water activity. Carvalho et al. [47] showed that the porous structure of the dried material ensures rapid dissolution of the product, which greatly facilitates its subsequent hydration as a food ingredient.

### 3.2. Composition, Bulk Density, and Water Activity of Raw and Reconstituted Mare’s Milk

The results showed that freeze-drying and reconstitution did not change the proportion between the milk components included in the dry matter (Table 1). In analyzing the main components of raw and reconstituted mare’s milk, no significant differences were found (*p* > 0.05). The ratio between non-fat solids and total protein in both the raw milk and reconstituted freeze-dried mare’s milk was 3.7. Additionally, the ratio of total protein to lactose in both samples was 0.4.

The average chemical composition of the mare’s milk, according to ref. [48,49] included a low amount of fat and proteins and a high amount of lactose. The report showed that the milk of Polish Coldblood mares in mid lactation contained 84.4 g/kg of solids—not-fat, 15.1 g/kg of fat, 65.3 g/kg of lactose, and 24.2 g/kg of total protein (14.6 g/kg of casein and 9.5 g/kg of whey protein) [48].

The only difference between the raw and reconstituted mare’s milk was the density value (Table 1). The reconstituted mare’s milk was characterized by a higher value (*p* < 0.05). This may be due to the freeze-drying process causing structural modifications in the micelles, which changes the interfacial properties.

### 3.3. Characteristics of Foam Formed from Raw and Reconstituted Freeze-Dried Mare’s Milk

The foaming capacity is the ability of a solution to bind air, which reached a value of 101.8 (raw milk) and 111.3% (reconstituted freeze-dried mare’s milk); hence, the foaming capacity of mare’s milk was very poor (Table 2). It is known that greater foaming is related to the rate at which proteins adsorb on the interfacial surface. Therefore, the more protein, the thicker the film layer at the interface. However, in our case, the foam produced was unstable. After 30 min, the generated foam was only 0.16% of its initial value in reconstituted freeze-dried mare’s milk. The conducted analysis showed that the rapid disappearance of the foam occurred within the first 5 min after beating. Raw mare’s milk gave no foam (FS = 0.03%). The foam produced immediately after beating from reconstituted freeze-dried mare’s milk was half as light as the solution, giving an FSW value of 106.3%. The proportion of the air fraction (Φ = 0.511%) in the foam from this milk was related to the slow phase transition from viscous solutions to semi-solid foam structures. This was due to the non-retention of air in the foam (wet rather than dry foam). This tendency is intensified for the proportion of the air fraction in the foam from raw milk (Φ = 0.781%, *p* < 0.05), even though there was very little of it.

According to reports, a colloidal dispersion is when the gas is dispersed continuously in the liquid phase [50]. Foam falls as a result of the loss of liquid from the spaces between the membranes owing to gravity, centrifugation, pressure, and evaporation, as well as the transportation of gas from smaller to larger bubbles, and the merging of bubbles after the destruction of the membranes. Through the comparison of the properties of the foam obtained with the content and type of protein in the milk, it was found that the effectiveness of the predominant whey protein in freeze-dried mare’s milk was limited in the stabilization and building up the foam. Rouimi et al. [51] investigated the foam stability and interfacial properties of milk, where different behaviors of whey proteins and caseins were observed. Additionally, the high surface elasticity of the aerated system was needed to inhibit rapid foam destabilization. This stems from the elasticity that whey proteins possess in contrast to micellar caseins, which did not exhibit any viscoelastic behavior at the interfaces [51]. Martínez-Padilla et al. [52] also obtained very stable foams from reconstituted skim milk powder fortified with whey protein concentrate.

In our study, we expected to obtain a more efficient and stable foam owing to the low-fat content of the solution. Kamath et al. [53] showed that the foam from skimmed milk was more stable than that from whole milk, where the bubbles were small and had a higher degree of rupture. To improve the foam properties of freeze-dried mare’s milk, the change of acidity in the starting solution must be analyzed. According to Yang and Zhao [33], a change in pH towards alkaline promotes stronger intramolecular electrostatic repulsions, as well as exposing hydrophobic amino acid residues. Hence, research on the foaming temperature should be the next step to improve foam performance and stability. Borcherding et al. [54] showed that an increase in the whipping temperature from 4 to 60 °C improved the stability of skimmed milk powder foam. Heating during the whipping process reduced the diameter of the bubbles and increased the density of the foam. Moreover, foam from unheated milk compared to foam from UHT or HT milk was more dense and had a smaller bubble diameter [54]. Furthermore, heating led to the exposure of functional groups from inside, or aggregation [55].

### 3.4. Ability of Raw and Reconstituted Freeze-Dried Mare’s Milk to Form Emulsions

The oil-binding capacity in raw and reconstituted freeze-dried mare’s milk was 2.19 and 2.20 g/g protein, respectively (*p* > 0.05, Table 3). The oil-binding capacity is an indicator of how much oil is bound and retained in the sample matrix by proteins. In this study, whey proteins were primarily responsible for the low value. It is known that mare’s milk has a relatively low casein content compared to the whey protein content. The proteins with lower water solubility are known to have a greater ability to bind to fat [56]. The oil-binding capacity value of raw and reconstituted freeze-dried mare’s milk (mean 2.2 g/g protein) was slightly lower than that of native whey protein isolate (WPI, 2.25 g/g protein) and enzymatically modified WPI (2.56 and 2.31 g/g protein), as determined by Yang and Zhao [33]. Other researchers determined the oil-binding capacity for milk protein concentrate (MPC60) to be 2.8 g/g protein [57], for cow’s milk it was 3.38 g/g protein [45], and for buffalo’s milk it was 5.18 g/g protein [58]. The evaluation of colloidal dispersions formed from freeze-dried mare’s milk is shown in Table 3. EAI and ESI indices were determined to quantify the ability of emulsifiers to stabilize O/W emulsions. Comparing the emulsifying activity index value, raw and reconstituted freeze-dried mares milk did not differ (EAI between 41.5 and 42.5 m^2^/g protein, *p* > 0.05). However, the emulsion stability index of reconstituted milk (ESI = 56.0 min) was greater than that of raw milk (ESI = 53.0 min, *p* < 0.05). Liu et al. [59] analyzed the effect of different emulsifiers on the properties and stability of casein-based emulsions. EAI values ranged from 12.62 to 17.54 m^2^/g, and the most stable emulsion had an ESI of 265.54 min. Silva et al. [60] showed that emulsions stabilized with WPI had a higher initial EAI value (~30 m^2^/g) than those with a casein to whey ratio of 50:50 (EAI ~1 m^2^/g). McClements et al. [61] found that the more stable the emulsion, the ESI tends towards infinity, while smaller values indicate an unstable emulsion. The same type of correlation was also observed by Liu et al. [59], where during the emulsion forming process, the protein molecules present in the solution diffused at the oil–water interface and formed a uniform layer around the oil droplets. This ability has been used in many food products due to emulsions and suspensions being typical systems [50]. According to Shah et al. [62], the emulsifying capacity of a protein solution was related to the ability of the protein molecules to reduce the surface tension at the oil–water interface. In addition, an analysis of the type of protein by Braun et al. [63] revealed more stable emulsions with casein, which was hydrophobic compared with hydrophilic whey proteins. Hence, the freeze-dried emulsion parameters of mare’s milk could be less favorable than those from solutions with a higher proportion of casein. Additionally, in the future, it is worth investigating the pH dependency of emulsion parameters. Khalesi and FitzGerald [57] showed that at a milk protein concentrate at pH 2, the EAI value was 42 m^2^/g, whereas at pH 10, the EAI value increased to 58 m^2^/g.

### 3.5. Structure of Freeze-Dried Mare’s Milk and Its Foam

**Figure 1:**
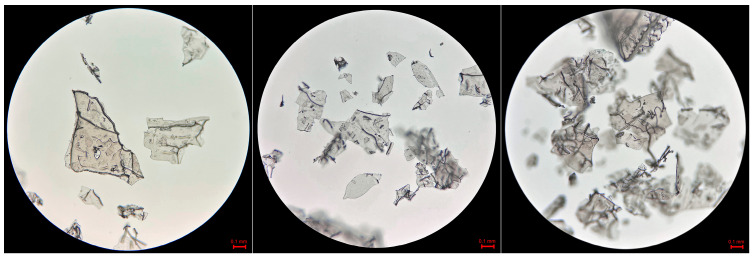
Microstructures of freeze-dried mare’s milk (optical microscopy, 100× objective).

**Figure 2:**
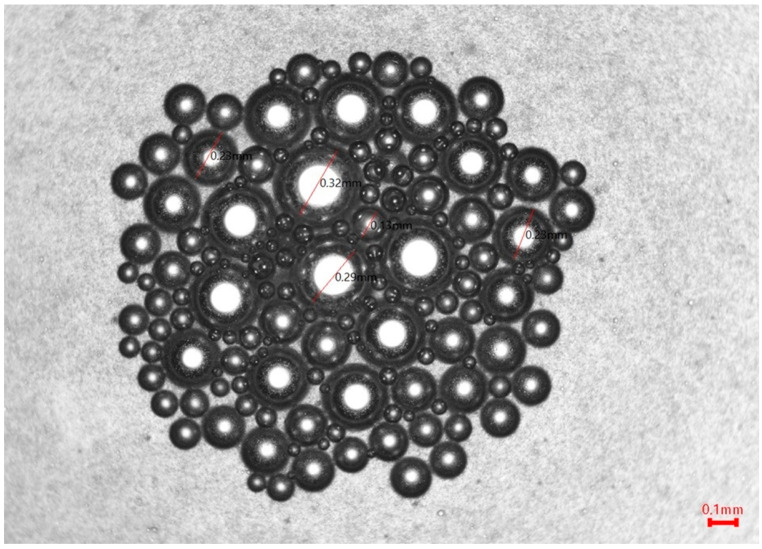
Microstructures of foam formed from reconstituted freeze-dried mare’s milk, (optical microscopy, 4× objective).

The microscopy images recorded for freeze-dried mare’s milk indicated their breakable internal structure and high fragmentation (Figure 1). However, photographs of the foam produced from reconstituted milk (Figure 2) confirmed a low capacity to swell and form a stable foam. The formed bubbles were small in diameter, with no bridges between them, and had regular round-shaped bubbles (spherical). There were no polyhedral-shaped bubbles. Furthermore, the bubbles were arranged loosely. Approx. 82% of the vesicles had a Ø of less than 0.14 mm, and only a few vesicles appeared bigger, with the largest possessing a Ø of 0.32. Thus, this was a more monodisperse than a polydisperse system.

### 3.6. Health Value of Raw and Reconstituted Freeze-Dried Mare’s Milk Fat

The calculated AI and TI for reconstituted milk was 1.02 and 0.53, respectively (Table 4). They were similar to raw mare’s milk (*p* > 0.05). In comparison, for cow’s milk at different months of lactation, the AI varied from 4.08 to 5.13 [64]. In general, AI describes fatty acids’ atherogenic potential, and TI shows the propensity for blood vessel thrombus forming [65]. Both indices have an important role and are used to determine the risk of various diseases, particularly coronary heart disease and cancer. It is known that the lower the indices values, the less risk of atherosclerosis [3]. The data presented in Table 4 show that the hypocholesterolemic fatty acid index (HcFA) of reconstituted freeze-dried mare’s milk was P_5_–P_95_ from 24.15 to 25.87 and the content of the required DFA was higher (53.67) than that of undesirable OFA (45.64), with an index average rating of 1.18. The obtained results suggested that mare’s milk fat was suitable for older people, who generally have various health risks. Therefore, in the future, it is important to consider the consequences of fatty acid composition on the nutritional value of the diet and, consequently, on health.

## 4. Conclusions

Herein, the use of the freeze-drying process for the preservation of mare’s milk has been demonstrated. A very low value of water activity and at the same time a high structural homogeneity of freeze-dried mare’s milk indicate a loose and poorly packed matrix. Therefore, the porous structure of the dried material, flowability, and lightness of freeze dried-mare’s milk ensure its quick dissolution, which greatly facilitates its subsequent hydration as a food ingredient. Freeze-dried mare’s milk after reconstitution retains the same proportion of protein in solid non-fat as in raw milk. Despite the similar content of basic ingredients in raw milk and in reconstituted freeze-dried mare’s milk, significant differences were found in the efficiency and parameters of the produced foam. Unlike raw mare’s milk, reconstituted freeze-dried mares milk produced foam. However, this foam was unstable, short-lived, and lacked the ability to retain air fractions. When analyzing the ability of milk to form emulsions, it was shown that the emulsifying activity index for both types of milk did not differ, while the emulsion stability index of reconstituted freeze-dried mare’s milk was higher than that of raw milk. Thus, it can be assumed that the freeze-drying process improves the binding degree and retention of oil by milk proteins.

The gained observation provides new opportunities in the processing of mare’s milk and may lead to novel products based on freeze-dried mare’s milk. Freeze-dried mare’s milk has limited use for the production of highly aerated foods, but forms emulsions comparable to milk protein concentrate. Our complete insight into reconstituted milk contributes to the development of new products from mare’s milk, while changing the supply profile of mare’s milk on the market. A limitation of the study is the continuous supply of mare’s milk to the freeze-dryers due to the volume of milk supplied and/or even its complete absence during the foaling period.

## Figures and Tables

**Table 1:** Gross composition and physicochemical characteristics of raw and reconstituted mare’s milk.

	Raw Mare’s Milk			Reconstituted Freeze-Dried Mare’s Milk		
Parameters	Mean	P_5_	P_95_	Mean	P_5_	P_95_
Solids—not-fat, g/kg	88.3 ^a^	88.3	88.3	88.4 ^a^	88.0	88.6
Fat, g/kg	17.2 ^a^	17.2	17.3	17.4 ^a^	16.2	18.1
Moisture, g/kg	898.5 ^a^	898.1	898.8	897.3 ^a^	888.9	905.7
Total protein ^1^, g/kg	23.8 ^a^	23.6	24.1	23.7 ^a^	22.6	24.8
Casein ^2^, g/kg	12.8 ^a^	12.6	13.0	12.5 ^a^	12.2	12.8
Whey protein ^3^, g/kg	10.9 ^a^	10.7	11.1	10.8 ^a^	10.3	11.0
Lactose, g/kg	63.2 ^a^	63.0	63.4	62.3 ^a^	60.6	64.1
Ash, g/kg	5.8 ^a^	5.6	6.1	5.6 ^a^	5.5	5.8
Solids—not-fat/total protein	3.7 ^a^			3.7 ^a^		
Total protein/lactose	0.4 ^a^			0.4 ^a^		
Density in 20 °C, g/mL	1.036 ^a^	1.034	1.039	1.039 ^b^	1.038	1.040
Water activity, -	0.9901 ^a^	0.9895	0.9907	0.9896 ^a^	0.9883	0.9908

^1^ Total protein = (total N − NPN) × 6.38; ^2^ casein = (total N − non-casein N − NPN) × 6.38; ^3^ whey protein = (non-casein N − NPN) × 6.38; P_5_–P_95_: confidence interval of the mean (*n* = 6); ^a, b^ different small letters in the superscript in rows indicate statistically significant differences at the level *p* = 0.05.

**Table 2:** Efficiency and parameters of the foam formed from raw and reconstituted freeze-dried mare’s milk.

	Raw Mare’s Milk			Reconstituted Freeze-Dried Mare’s Milk		
Parameters	Mean	P_5_	P_95_	Mean	P_5_	P_95_
FP, %	101.8 ^a^	100.3	103.4	111.3 ^b^	109.6	113.0
FS, %	0.03 ^a^	−0.05	0.12	0.16 ^b^	−0.26	0.59
FSW, %	352.1 ^b^	344.0	361.1	106.3 ^a^	103.5	109.1
Φ, %	0.781 ^b^	0.699	0.782	0.511 ^a^	0.500	0.522

P_5_–P_95_: confidence interval of the mean (*n* = 6). FP—foam performance; FS—foam stability, FSW—foam swelling, Φ—proportion of air fraction in foam. ^a, b^ Different small letters in the superscript in rows indicate statistically significant differences at the level *p* = 0.05.

**Table 3:** Properties of emulsions based on raw and reconstituted freeze-dried mare’s milk.

	Raw Mare’s Milk			Reconstituted Freeze-Dried Mare’s Milk		
Parameters	Mean	P_5_	P_95_	Mean	P_5_	P_95_
Oil-binding capacity, g/g protein	2.20 ^a^	2.06	2.48	2.19 ^a^	2.12	2.26
EAI, m^2^/g	41.5 ^a^	39.2	43.6	42.5 ^a^	41.1	43.9
ESI, min	53.0 ^a^	51.1	54.0	56.0 ^b^	53.3	58.6

P_5_–P_95_: confidence interval of the mean (*n* = 6). EAI—emulsifying activity index, ESI—emulsion stability index. ^a, b^ Different small letters in the superscript in rows indicate statistically significant differences at the level *p* = 0.05.

**Table 4:** Health-promoting properties of raw and reconstituted freeze-dried mare’s milk fat.

	Raw Mare’s Milk			Reconstituted Freeze-Dried Mare’s Milk		
Parameters	Mean	P_5_	P_95_	Mean	P_5_	P_95_
AI	1.01 ^a^	0.98	1.05	1.02 ^a^	0.98	1.07
TI	0.51 ^a^	0.43	0.59	0.53 ^a^	0.46	0.60
HcFA	25.07 ^a^	24.28	25.86	25.01 ^a^	24.15	25.87
DFA	53.64 ^a^	52.60	54.68	53.67 ^a^	52.62	54.71
OFA	45.63 ^a^	44.57	46.69	45.64 ^a^	44.57	46.71
DFA/OFA	1.18 ^a^			1.18 ^a^		

P_5_–P_95_: confidence interval of the mean (*n* = 6). AI = atherogenic index, AI = (C12:0 + 4 × C14:0 + C16:0)/UFA. TI = thrombogenic index, TI = (C14:0 + C16:0 + C18:0)/[(0.5 x MUFA) + (3 × n-3) + (0.5 × n-6) + (n-3/n-6)]. HcFA: hypercholesterolemic fatty acid index, HcFA = C14:0 + C16:0. DFA = Σ UFA + C18:0. OFA = Σ SFA − C18:0. Where Σ UFA is sum of MUFA (C10:1, C12:1, C14:1 cis-9, C16:1 cis-9, C16:1 trans-9, C17:1 cis-9, C18:1 cis-9, C18:1 cis-11, C18:1 cis-12, C18:1 trans-9, C18:1 trans-11, and C20:1 cis-11) and PUFA (C18:2 cis-9,trans-11, C18:2 cis-9,cis-12, C18:3 cis-6,cis-9,cis-12, C18:3 cis-9,cis-12,cis-15, C20: 2n-6, C20: 3n-6, C20: 4n-6, C20: 5n-3, and C22: 5n-3); Σ SFA is sum of C4:0, C6:0; C8:0, C10:0, C11:0, C12:0, C13:0, C14:0, C14:0 iso, C15:0, C15:0 iso, C15:0 anteiso, C16:0, C16:0 iso, C17:0, C17:0 iso, C17:0 anteiso, C18:0, C18:0 iso, C20:0, C22:0, and C24:0. ^a^ Different small letters in the superscript in rows indicate statistically significant differences at the level *p* = 0.05.

## Data Availability

Data is contained within the article.

## References

[1] Musaev A., Sadykova S., Anambayeva A., Saizhanova M., Balkanay G., Kolbaev M. (2021). Mare’s milk: Composition, properties, and application in medicine. Arch. Razi Inst..

[2] Kushugulova A., Kozhakhmetov S., Sattybayeva R., Nurgozhina A., Ziyat A., Yadav H., Marotta F. (2018). Mare’s milk as a prospective functional product. Funct. Foods Health Dis..

[3] Teichert J., Cais-Sokolińska D., Bielska P., Danków R., Chudy S., Kaczyński Ł.K., Biegalski J. (2021). Milk fermentation affects amino acid and fatty acid profile of mare milk from Polish Coldblood mares. Int. Dairy J..

[4] Khajeh E., Jamshidian-Mojaver M., Naeemipour M., Farzin H. (2021). The identification of a novel peptide derived from lactoferrin isolated from camel milk with potential antimicrobial activity. Iran. J. Med. Microbiol..

[5] Cosentino C., Labella C., Elshafie H.S., Camele I., Musto M., Paolino R., D’Adamo C., Freschi P. (2016). Effects of different heat treatments on lysozyme quantity and antimicrobial activity of jenny milk. J. Dairy Sci..

[6] Narmuratova Z., Hentati F., Girardet J.M., Narmuratova M., Cakir-Kiefer C. (2022). Equine lactoferrin: Antioxidant properties related to divalent metal chelation. LWT.

[7] Campione E., Cosio T., Rosa L., Lanna C., Girolamo S., Di Gaziano R., Valenti P., Bianchi L. (2020). Lactoferrin as protective natural barrier of respiratory and intestinal mucosa against coronavirus infection and inflammation. Int. J. Mol. Sci..

[8] Kaić A., Luštrek B., Simčič M., Potočnik K. (2019). Milk quantity, composition and hygiene traits of routinely machine milked lipizzan mares. Slov. Vet. Res..

[9] Miraglia N., Salimei E., Fantuz F. (2020). Equine milk production and valorization of marginal areas—A review. Animals.

[10] Ciurzynska A., Lenart A. (2011). Freeze-drying—Application in food processing and biotechnology—A review. Pol. J. Food Nutr. Sci..

[11] Bhatta S., Stevanovic Janezic T., Ratti C. (2020). Freeze-drying of plant-based foods. Foods.

[12] Doneva M.D., Dyankova S.M., Miteva D.P., Nacheva I.B., Metodieva P.M. (2021). Cryobiological studies and freeze drying of cow’s milk and curd. J. Chem. Technol. Meta..

[13] Ratti C. (2001). Hot air and freeze-drying of high-value foods: A review. J. Food Eng..

[14] Martysiak-Żurowska D., Rożek P., Puta M. (2022). The effect of freeze-drying and storage on lysozyme activity, lactoferrin content, superoxide dismutase activity, total antioxidant capacity and fatty acid profile of freeze-dried human milk. Dry. Technol..

[15] Shukla S. (2011). Freeze drying process: A review. Int. J. Pharm. Sci..

[16] Gaidhani K.A., Harwalkar M., Bhambere D., Nirgude P.S. (2015). Lyophilization/freeze drying—A review. World J. Pharm. Res..

[17] Nowak D., Jakubczyk E. (2020). The freeze-drying of foods-The characteristic of the process course and the effect of its parameters on the physical properties of food materials. Foods.

[18] Oyinloye T.M., Yoon W.B. (2020). Effect of freeze-drying on quality and grinding process of food produce: A review. Processes.

[19] Tastemirova U., Ciprovica I., Shingisov A. (2020). The comparison of the spray-drying and freeze-drying techniques for camel milk: A review. Livest. Res. Rural Dev..

[20] Myrkalykov B., Shingisov A., Ospanov A., Simov Z., Latif A., Aripbaeva A. (2018). Freeze drying of sheep milk: Calculating process duration. Eur. J. Soc. Sci..

[21] Zhang Y., Zheng Z., Liu C., Tan C., Xie K., Liu Y. (2022). A comparative study between freeze-dried and spray-dried goat milk on lipid profiling and digestibility. Food Chem..

[22] Tastemirova U., Mukhtarkhanova R., Alimardanova M., Alibekov R., Shingisov A. (2022). Impact of vacuum freeze-drying on the reconstituted camel milk composition. Food Sci. Technol..

[23] Polidori P., Spera M.D., Sabatini A., Vincenzetti S. (2019). Comparison of nutritional characteristics of fresh and freeze-dried donkey milk. Food Sci. Nutr. Technol..

[24] Teichert J., Cais-Sokolińska D., Danków R., Pikul J., Chudy S., Bierzuńska P., Kaczyński Ł.K. (2020). Color stability of fermented mare’s milk and a fermented beverage from cow’s milk adapted to mare’s milk composition. Foods.

[25] Kondybayev A., Konuspayeva G., Strub C., Loiseau G., Mestres C., Grabulos J., Manzano M., Akhmedsadykowa S., Achir N. (2022). Growth and metabolism of *Lacticaseibacillus casei* and *Lactobacillus kefiri* isolated from qymyz, a traditional fermented central asian beverage. Fermentation.

[26] Yoo S.R., Lee S.W., Jeon H.M. (2020). The role of customer experience, food healthiness, and value for revisit intention in Grocerant. Sustainability.

[27] Harizi N., Madureira J., Zouari A., Ayadi M.A., Cabo Verde S., Boudhrioua N. (2023). Effects of spray drying, freeze drying and gamma irradiation on the antioxidant activities of camel and cow milk fractions. Processes.

[28] Kim S.H., Chang Y.H., Kwak H.S. (2010). Physicochemical properties of reconstituted milk made from freeze-dried milk powder or spray-dried milk powder. Korean J. Food Sci. Ani. Resour..

[29] Zou Z., Duley J.A., Cowley D.M., Reed S., Arachchige B.J., Bhandari B., Shaw P.N., Bansal N. (2022). Physicochemical properties and whey proteomes of camel milk powders produced by different concentration and dehydration processes. Foods.

[30] Castro-Albarran J., Aguilar Uscanga B., Calon F., St-Amour I., Solis J., Saucier L., Ratti C. (2016). Spray and freeze drying of human milk on the retention of immunoglobulins (IgA, IgG, IgM). Dry. Technol..

[31] (2008). Milk and Milk Products—Guidance on Sampling.

[32] Polidori P., Cammertoni N., Santini G., Klimanova Y., Zhang J.-J., Vincenzetti S. (2021). Nutritional properties of camelids and equids fresh and fermented milk. Dairy.

[33] Yang Y.F., Zhao X.H. (2022). Structure and property changes of whey protein isolate in response to the chemical modification mediated by horseradish peroxidase, glucose oxidase and d-glucose. Food Chem..

[34] Alamprese C., Rollini M., Musatti A., Ferranti P., Barbiroli A. (2022). Emulsifying and foaming properties of a hydrophobin-based food ingredient from *Trichoderma reesei*: A phenomenological comparative study. LWT.

[35] Sun Y., Yu X., Hussain M., Li X., Liu L., Liu Y., Jiang S. (2022). Influence of milk fat globule membrane and milk protein concentrate treated by ultrasound on the structural and emulsifying stability of mimicking human fat emulsions. Ultrason. Sonochem..

[36] Chang Y., Hartel R.W. (2002). Measurement of air cell distributions in dairy foams. Int. Dairy J..

[37] Cais-Sokolińska D., Pikul J., Wójtowski J., Danków R., Teichert J., Czyżak-Runowska G., Bagnicka E. (2015). Evaluation of quality of kefir from milk obtained from goats supplemented with a diet rich in bioactive compounds. J. Sci. Food Agric..

[38] Kara K. (2020). Milk urea nitrogen and milk fatty acid compositions in dairy cows with subacute ruminal acidosis. Vet. Med..

[39] Ulbricht T.L.V., Southgate D.A.T. (1991). Coronary heart disease: Seven dietary factors. Lancet.

[40] Pilarczyk R., Wójcik J., Sablik P., Czerniak P. (2015). Fatty acid profile and health lipid indices in the raw milk of simmental and holstein-friesian cows from an organic farm. S. Afr. J. Anim. Sci..

[41] Mazur-Kuśnirek M., Antoszkiewicz Z., Lipiński K., Kaliniewicz J., Kotlarczyk S. (2019). The effect of polyphenols and vitamin E on the antioxidant status and meat quality of broiler chickens fed low-quality oil. Arch. Anim. Breed..

[42] Sulieman A.M.E., Elamin O.M., Elkhalifa E.A., Laleye L. (2017). Utilization of differential scanning calorimetry (DSC) in differentiation between cow milk and camel milk powder. EC Nutr..

[43] Meena G.S., Singh A.K., Arora S., Borad S., Sharma R., Gupta V.K. (2017). Physico-chemical, functional and rheological properties of milk protein concentrate 60 as affected by disodium phosphate addition, diafiltration and homogenization. J. Food Sci. Technol..

[44] Brożek O.M., Kiełczewska K., Bohdziewicz K. (2022). Fatty acid profile and thermal characteristics of ovine and bovine milk and their mixtures. Int. Dairy J..

[45] Alves E.S., Ferreira C.S.R., Souza P.R., Bruni A.R.S., Castro M.C., Saqueti B.H.F., Santos O.O., Madrona G.S., Visentainer J.V. (2023). Freeze-dried human milk microcapsules using gum arabic and maltodextrin: An approach to improving solubility. Int. J. Biol. Macromol..

[46] Öztürk H.İ. (2022). The effect of different lyophilisation pressures on the microbiological stability, physicochemical, microstructural, and sensorial properties of yoghurt powders. Int. Dairy J..

[47] Carvalho M.J., Perez-Palacios T., Ruiz-Carrascal J. (2017). Physico-chemical and sensory characteristics of freeze-dried and air-dehydrated yogurt foam. LWT.

[48] Cais-Sokolińska D., Danków R., Bierzuńska P., Kaczyński Ł.K., Chudy S., Teichert J., Pikul J. (2018). Freezing point and other technological properties of milk of the Polish Coldblood horse breed. J. Dairy Sci..

[49] Cais-Sokolińska D., Wójtowski J., Pikul J. (2016). Rheological, texture and sensory properties of kefir from mare’s milk and its mixtures with goat and sheep milk. Mljekarstvo.

[50] Vilela A., Cosme F., Pinto T. (2018). Emulsions, foams, and suspensions: The microscience of the beverage industry. Beverages.

[51] Rouimi S., Schorsch C., Valentini C., Vaslin S. (2005). Foam stability and interfacial properties of milk protein–surfactant systems. Food Hydrocoll..

[52] Martínez-Padilla L.P., García-Mena V., Casas-Alencáster N.B., Sosa-Herrera M.G. (2014). Foaming properties of skim milk powder fortified with milk proteins. Int. Dairy J..

[53] Kamath S., Huppertz T., Houlihan A.V., Deeth H.C. (2008). The influence of temperature on the foaming of milk. Int. Dairy J..

[54] Borcherding K., Lorenzen P.C., Hoffmann W., Schrader K. (2008). Effect of foaming temperature and varying time/temperature-conditions of pre-heating on the foaming properties of skimmed milk. Int. Dairy J..

[55] Einhorn-Stoll U., Ulbrich M., Sever S., Kunzek H. (2005). Formation of milk protein–pectin conjugates with improved emulsifying properties by controlled dry heating. Food Hydrocoll..

[56] Priyanka V., Shilpashree B.G., Ashwini A. (2022). Physico-chemical and techno-functional attributes of dairy powders. Int. Res. J. Mod. Eng. Technol. Sci..

[57] Khalesi M., FitzGerald R.J. (2022). Impact of variation in calcium level on the technofunctional properties of milk protein concentrate. Colloids Surf. A Physicochem. Eng. Asp..

[58] Patil A.T., Meena G.S., Upadhyay N., Khetra Y., Borad S., Singh A.K. (2018). Production and characterization of milk protein concentrates 60 (MPC60) from buffalo milk. LWT.

[59] Liu Y., Wei Z.C., Deng Y.Y., Dong H., Zhang Y., Tang X.J., Zhang M.W. (2020). Comparison of the effects of different food-grade emulsifiers on the properties and stability of a casein-maltodextrin-soybean oil compound emulsion. Molecules.

[60] Silva M., Zisu B., Chandrapala J. (2020). Interfacial and emulsification properties of sono-emulsified grape seed oil emulsions stabilized with milk proteins. Food Chem..

[61] McClements D.J., Lu J., Grossmann L. (2022). Proposed methods for testing and comparing the emulsifying properties of proteins from animal, plant, and alternative sources. Colloid Interface Sci..

[62] Shah K., Salunke P., Metzger L. (2022). Effect of storage of skim milk powder, nonfat dry milk and milk protein concentrate on functional properties. Dairy.

[63] Braun K., Hanewald A., Vilgis T.A. (2019). Milk emulsions: Structure and stability. Foods.

[64] Nantapo C.T.W., Muchenje V., Hugo A. (2014). Atherogenicity index and health-related fatty acids in different stages of lactation from Friesian, Jersey and Friesian × Jersey cross cow milk under a pasture-based dairy system. Food Chem..

[65] Chen J., Liu H. (2020). Nutritional indices for assessing fatty acids: A mini-review. Int. J. Mol. Sci..

